# Incremental versus conventional haemodialysis in end-stage kidney disease: a systematic review and meta-analysis

**DOI:** 10.1093/ckj/sfad280

**Published:** 2023-11-08

**Authors:** Kullaya Takkavatakarn, Kavita Jintanapramote, Jeerath Phannajit, Kearkiat Praditpornsilpa, Somchai Eiam-Ong, Paweena Susantitaphong

**Affiliations:** Division of Nephrology, Department of Medicine, King Chulalongkorn Memorial Hospital, Faculty of Medicine, Chulalongkorn University, Bangkok, Thailand; Division of Nephrology, Department of Medicine, Bhumibol Adulyadej Hospital, Royal Thai Air Force, Bangkok, Thailand; Division of Nephrology, Department of Medicine, King Chulalongkorn Memorial Hospital, Faculty of Medicine, Chulalongkorn University, Bangkok, Thailand; Research Unit for Metabolic Bone Disease in CKD patients, King Chulalongkorn Memorial Hospital, Faculty of Medicine, Chulalongkorn University, Bangkok, Thailand; Division of Nephrology, Department of Medicine, King Chulalongkorn Memorial Hospital, Faculty of Medicine, Chulalongkorn University, Bangkok, Thailand; Division of Nephrology, Department of Medicine, King Chulalongkorn Memorial Hospital, Faculty of Medicine, Chulalongkorn University, Bangkok, Thailand; Division of Nephrology, Department of Medicine, King Chulalongkorn Memorial Hospital, Faculty of Medicine, Chulalongkorn University, Bangkok, Thailand; Research Unit for Metabolic Bone Disease in CKD patients, King Chulalongkorn Memorial Hospital, Faculty of Medicine, Chulalongkorn University, Bangkok, Thailand

**Keywords:** conventional haemodialysis, incremental haemodialysis, once-weekly haemodialysis, residual kidney function, twice-weekly haemodialysis

## Abstract

**Background:**

Appropriate dialysis prescription in the transitional setting from chronic kidney disease to end-stage kidney disease is still challenging. Conventional thrice-weekly haemodialysis (HD) might be associated with rapid loss of residual kidney function (RKF) and high mortality. The benefits and risks of incremental HD compared with conventional HD were explored in this systematic review and meta-analysis.

**Methods:**

We searched MEDLINE, Scopus and Cochrane Central Register of Controlled Trials up to April 2023 for studies that compared the impacts of incremental (once- or twice-weekly HD) and conventional thrice-weekly HD on cardiovascular events, RKF, vascular access complications, quality of life, hospitalization and mortality.

**Results:**

A total of 36 articles (138 939 participants) were included in this meta-analysis. The mortality rate and cardiovascular events were similar between incremental and conventional HD {odds ratio [OR] 0.87 [95% confidence interval (CI)] 0.72–1.04 and OR 0.67 [95% CI 0.43–1.05], respectively}. However, hospitalization and loss of RKF were significantly lower in patients treated with incremental HD [OR 0.44 (95% CI 0.27–0.72) and OR 0.31 (95% CI 0.25–0.39), respectively]. In a sensitivity analysis that included studies restricted to those with RKF or urine output criteria, incremental HD had significantly lower cardiovascular events [OR 0.22 (95% CI 0.08–0.63)] and mortality [OR 0.54 (95% CI 0.37–0.79)]. Vascular access complications, hyperkalaemia and volume overload were not statistically different between groups.

**Conclusions:**

Incremental HD has been shown to be safe and may provide superior benefits in clinical outcomes, particularly in appropriately selected patients. Large-scale randomized controlled trials are required to confirm these potential advantages.

## Graphical Abstract

**Graphical Abstract:**
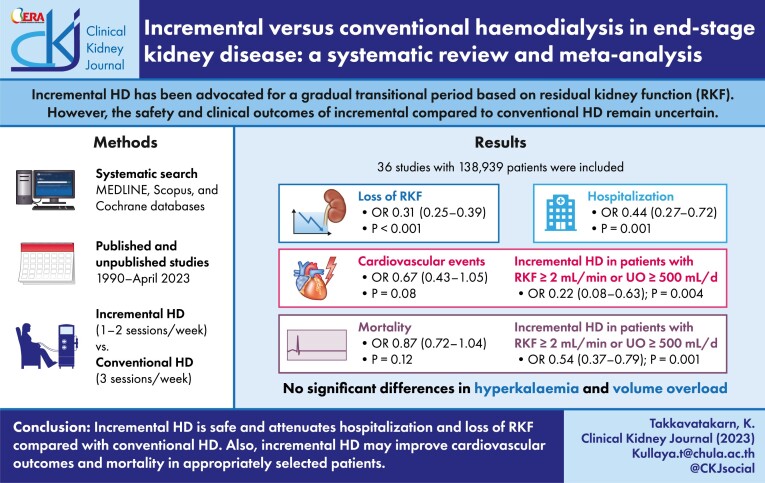

## INTRODUCTION

In the USA, >130 000 patients each year are newly diagnosed with end-stage kidney disease (ESKD) requiring haemodialysis (HD) [1]. Patients with ESKD are routinely initiated on a conventional thrice-weekly HD regimen as a standard HD prescription worldwide. Despite being a life-sustaining treatment, HD is a life-altering process with a high cost and is associated with marked declines in health-related quality of life. In addition, an abrupt transition from pre-dialysis to dialysis with thrice-weekly HD is associated with a significant increase in loss of residual kidney function (RKF) and mortality during the first 3 months [2, 3].

Incremental HD, with once- or twice-weekly HD as a means of allowing a gradual transition period, has recently been advocated. The dialysis dose of this approach is personalized according to RKF. Incremental HD provides a gentler start with less frequent HD and gradually increases the amount of dialysis as the RKF of the patient is progressively lost [4]. It has been suggested that incremental HD may have several benefits, including preserving RKF, lowering costs and improving quality of life compared with conventional treatment. Despite the several potential benefits of incremental HD, not all maintenance HD patients are suitable for this approach. The safety-related issues of incremental HD, including insufficient clearance of uraemic solutes, volume overload and hyperkalaemia, are still a major concern.

There is growing interest and an increasing number of reports of the benefits of incremental HD on RKF; however, most of these studies include a small number of patients and observational designs. Therefore, the actual effects of incremental HD on various outcomes remain inconclusive. To address these important issues, we aim to synthesize the available evidence on the safety and efficacy of incremental HD. This systematic review and meta-analysis was conducted to evaluate the impacts of incremental HD on cardiovascular events, RKF, vascular access complications, quality of life, hospitalization and mortality compared with conventional HD in ESKD patients.

## MATERIALS AND METHODS

### Data source and searches

This systematic review and meta-analysis was conducted in accordance with the Preferred Reporting Items for Systematic reviews and Meta-Analyses (PRISMA) statement. To identify eligible studies, a search was conducted in MEDLINE, Scopus and the Cochrane Central Register of Controlled Trials from 1990 to April 2023. Reference lists of the obtained articles were also searched for relevant publications. Also, unpublished data were sought from ClinicalTrials.gov and conference abstracts. The study protocol was registered with PROSPERO (CRD42022378215). For the search, the following terms were used: ‘incremental dialysis’ OR ‘conventional hemodialysis’ OR ‘once-weekly hemodialysis’ OR ‘twice-weekly hemodialysis’ OR ‘thrice-weekly hemodialysis’. The search was limited to the English language.

### Study selection

We included studies if they were either randomized controlled trials (RCTs), non-randomized trials or observational cohorts. In order to be eligible for inclusion, the studies had to meet the following criteria: an original article or intervention studies in humans with ESKD that compared the effects and/or safety of incremental and conventional HD in ESKD patients. The intervention of interest was incremental HD (once- or twice-weekly HD) compared with conventional HD (thrice-weekly HD). The outcomes included changes in urine output and quality of life before and after the intervention and clinical outcomes, comprising vascular access complications, volume overload, hyperkalaemia, cardiovascular events, hospitalization and mortality. Narrative reviews and case reports/series were excluded.

Two authors (K.T. and K.J.) separately screened the titles and abstracts of all electronic citations and obtained the full-text articles for the comprehensive review. Both authors then independently re-evaluated these articles. Disagreements were resolved through consensus and arbitration by the third author (P.S.).

### Data extraction

The study characteristics and outcomes of interest were independently reviewed by the authors. The data extracted included study authors, country, year of publication, number of patients, mean age, population characteristics and dialysis frequency (once-, twice- or thrice-weekly HD). The outcomes of interest that were extracted included urine output (ml/24 hours), quality of life and depression scores at baseline and at the end of the study. The incidence of death, hospitalization, cardiovascular events, loss of renal function, vascular access complications, volume overload and hyperkalaemia were also assessed.

### Quality assessment

Two reviewers (K.T. and K.J.) independently assessed the quality of the studies using the Newcastle–Ottawa Scale (NOS) for observational studies and the Cochrane Risk of Bias Tool (RoB 2) for RCTs.

The NOS consists of three quality domains: selection, comparability of the groups and outcome assessment. Each study received a score between 0 and 9, with a score ≥7 indicating high quality [5]. The RoB 2 tool includes five domains: randomization process, deviations from the intended interventions, missing outcome data, measurement of the outcome and selection of the reported result. The summary graphic was created using the RobVis22 application to show the independent domains for risk of bias. The studies were classified as ‘high risk,’ ‘some concerns’ or ‘low risk’ based on their scores [6].

### Data analysis

We used random effects model meta-analysis to compute the odds ratio (OR) in binary variables, including loss of RKF, vascular access and HD complications, hospitalization, cardiovascular events and mortality. To analyse continuous variables, we used the mean difference (MD) from baseline to the end of the study between incremental and conventional HD. All pooled estimates are presented with a 95% confidence interval (CI). Heterogeneity was assessed using the *I*^2^ index and the Q-test *P*-value, with an *I*^2^ index >75% indicating medium–high heterogeneity [7].

To identify possible effect modifiers on the pooled analyses, sources of heterogeneity were explored by subgroup analyses according to study design and patient characteristics. We conducted a sensitivity analysis to assess the consistency of the results. Statistical significance was determined when the *P*-value was <.05. Publication bias was formally assessed using funnel plots and the Egger test [8]. All analyses were performed using Comprehensive Meta-Analysis software version 2.0 (www.meta-analysis.com; Biostat, Englewood, NJ, USA). The forest plots were created by the ‘forestplot’ package in R software version 4.2.2 (R Foundation for Statistical Computing, Vienna, Austria).

## RESULTS

### Search results and characteristics of the studies

**Figure 1:**
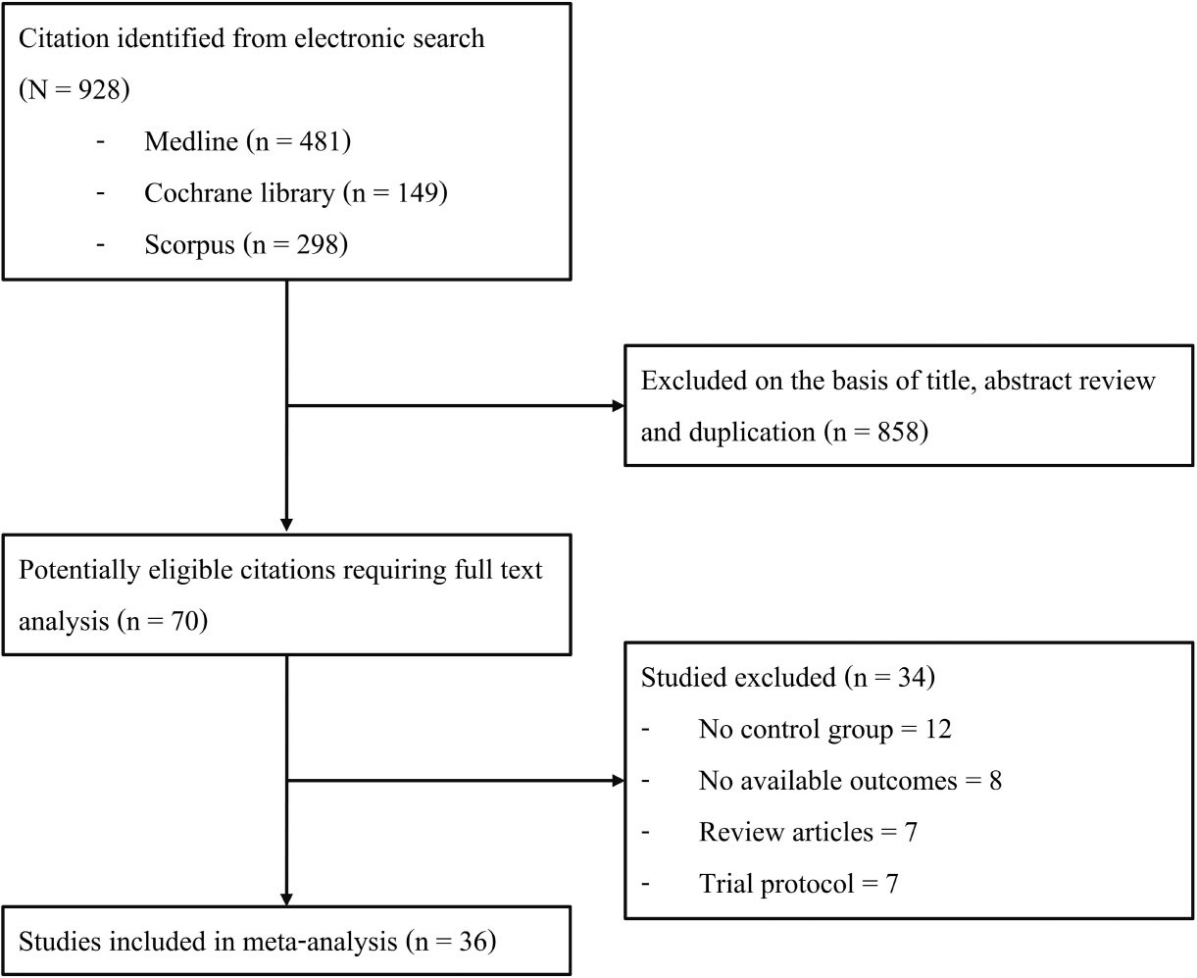
PRISMA flow diagram.

A total of 928 potentially relevant records were identified. The full-text screening was performed for 70 articles, and 36 studies [9–44] (35 officially published articles and 1 abstract) fulfilled the eligibility criteria. A flow diagram of the study selection is presented in Fig. 1.

The main characteristics of the studies are summarized in Table 1. The included studies were published from 1999 to June 2022. There were 138 939 patients with a mean age ranging from 43.8 to 70 years. Thirty-one studies were observational studies, four were RCTs and one was a non-randomized controlled study. Fifteen studies were performed in Asia, ten in Europe, seven in North America, two in Africa, one in South Africa and one in Australia.

**Table 1:** Characteristics of the studies included in the meta-analysis.

Author	Year	Country	Study design	Sample size	Age (years)	Incremental HD	Renal function or urine output in inclusion criteria	Quality
Hanson	1999	USA	Observational study	15 067	59.5	Twice-weekly HD		8^a^
Lin	2009	Taiwan	Observational study	74	66	Twice-weekly HD		6^a^
Supasyndh	2009	Thailand	Observational study	142	44.2	Twice-weekly HD		5^a^
Stankuvienė	2010	Lithuania	Observational study	2428	58	Once- or twice-weekly HD		5^a^
Fernandez-Lucas	2012	Spain	Observational study	95	62.5	Twice-weekly HD	KRU ≥2.5 ml/min	6^a^
Lin	2012	China	Observational study	2572	57.9	Twice-weekly HD		6^a^
Teruel-Briones	2013	Spain	Observational study	110	63.9	Twice-weekly HD	KRU ≥2.5 ml/min	9^a^
Bieber	2014	China	Observational study	1379	59.4	Twice-weekly HD		5^a^
Caria	2014	Italy	Prospective non-randomized controlled study	68	64.8	Once-weekly HD with low protein diet	eGFR 5–10 ml/min	7^a^
Cheng	2014	China	Observational study	143	61.7	Twice-weekly HD		6^a^
Lei	2014	China	Observational study	129	61.8	Twice-weekly HD		6^a^
Panaput	2014	Thailand	Observational study	673	55.2	Twice-weekly HD		7^a^
Zhang	2014	China	Observational study	85	56.8	Twice-weekly HD		8^a^
Hwang	2016	Korea	Observational study	685	58.4	Twice-weekly HD		7^a^
Mathew	2016	USA	Observational study	50 596	63.1	Once- or twice-weekly HD		8^a^
Obi	2016	USA	Observational study	8419	68	Twice-weekly HD		9^a^
Mukherjee	2017	India	Observational study	117	58.2	Twice-weekly HD		4^a^
Park	2017	Korea	Observational study	312	61.2	Once- or Twice-weekly HD		7^a^
Wang	2017	USA	Observational study	5561	70	Twice-weekly HD		7^a^
Lin	2018	China	Observational study	106	60.1	Twice-weekly HD		5^a^
Yan	2018	China	Observational study	1265	59.7	Twice-weekly HD		8^a^
Davenport	2019	UK	Observational study	709	64	Twice-weekly HD		6^a^
Kamal	2019	UK	Observational study	154	61.2	Twice-weekly HD	KRU ≥3 ml/min	9^a^
Shah (abstract)	2019	India	Observational study	19 455	52	Once- or twice-weekly HD		5^a^
Wolley	2019	Australia and New Zealand	Observational study	27 513	62.2	Once- or twice-weekly HD		8^a^
Chaker	2020	Tunisia	Observational study	88	56	Twice-weekly HD		4^a^
Dai	2020	China	RCT	140	50.9	Twice-weekly HD		Low risk^b^
Chen	2021	China	Observational study	113	59.7	Once- or twice-weekly HD		8^a^
Nieves-Anaya	2021	Mexico	Observational study	88	55.2	Twice-weekly HD		4^a^
Casino	2022	Italy	Observational study	202	66	Once- or twice-weekly HD	Urine output ≥500 ml/day	7^a^
Jaques	2022	Switzerland	Observational study	234	62	Twice-weekly HD	KRU ≥2 ml/min and urine output ≥500 ml/day	8^a^
Murea (1)	2022	USA	RCT	48	61.3	Twice-weekly HD	eGFR ≥5 ml/min/1.73 m^2^ and urine output ≥500 ml/day	Low risk^b^
Murea (2)	2022	USA	RCT	48	61.3	Twice-weekly HD	eGFR ≥5 ml/min/1.73 m^2^ and urine output ≥500 ml/day	Low risk^b^
Tawfik	2022	Egypt	Observational study	50	43.8	Twice-weekly HD	KRU >3 ml/min or urine output >500 ml/day	4^a^
Torreggiani	2022	France	Observational study	158	69.5	Once- or twice-weekly HD		8^a^
Vilar	2022	UK	RCT	55	62.2	Twice-weekly HD	KRU ≥3 ml/min/1.73 m^2^	Low risk^b^

a Newcastle–Ottawa Scale for assessing the quality of observational studies.

b ROB 2 for assessing the quality of RCTs.

eGFR: estimated glomerular filtration rate; KRU: renal urea clearance.

Incremental HD prescription in the enrolled studies referred to two HD sessions per week in 27 studies, one or two HD sessions per week in 8 studies and one HD session per week in combination with a very low protein diet in 1 study. There were RKF (urea clearance ranging from ≥2–≥3 ml/min) or urine output criteria (≥500 ml/day) for incremental HD enrolment in 10 studies.

### Risk-of-bias assessment

Table 1 summarizes the results of the risk of bias assessment of the included studies. The NOS scores of observational and non-randomized studies enrolled in this meta-analysis ranged from 5 to 8 (moderate to high quality). Four RCTs had a low risk of bias according to the Cochrane RoB tool.

### Patient characteristics

Serum haemoglobin, phosphate and albumin levels were comparable between patients treated with incremental and conventional HD. Serum β2-microglobulin (β2M) levels were significantly lower in patients with incremental compared with conventional HD [MD −11.18 μg/ml (95% CI −15.26 to −7.10), *P* < .001]. Patients with incremental HD had a significantly lower normalized protein catabolic rate (nPCR) than those with conventional HD [MD −0.07 (95% CI −0.12 to −0.23), *P* = .004]. There was no significant difference in single pool *Kt*/*V* between incremental and conventional HD. However, standardized *Kt*/*V* was significantly lower in incremental HD patients [MD −0.42 (95% CI −0.78 to −0.07), *P* = .02].

### Loss of residual kidney function and 24-hour urine output

**Figure 2:**
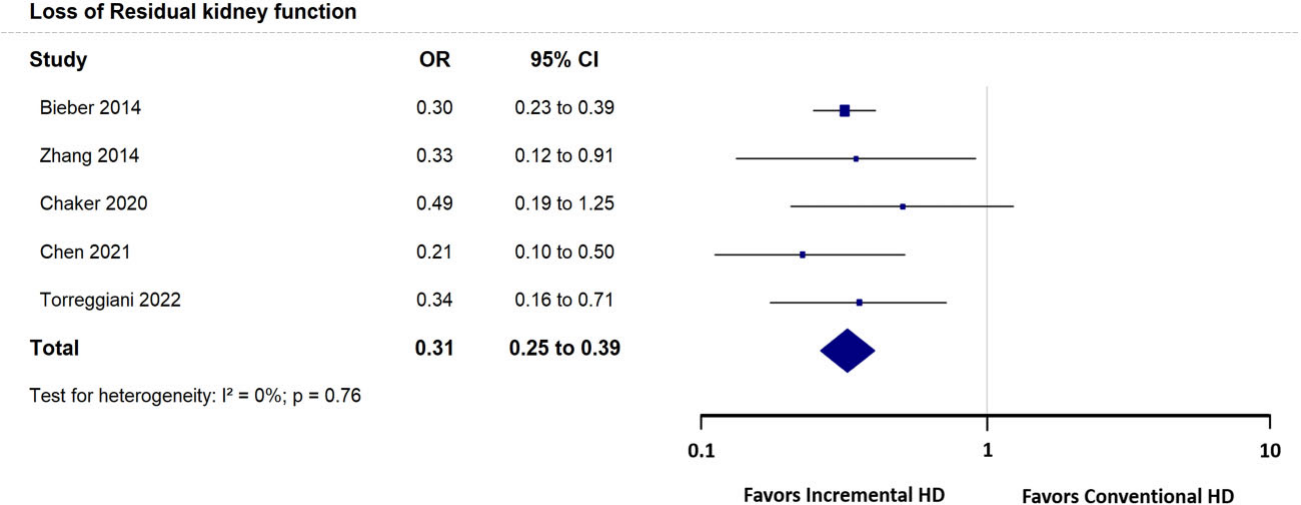
Loss of RKF forest plot.

Five studies [14, 16, 18, 32, 37] reported the incidence of loss of RKF defined by urine output <200 ml/day. Of these studies, incremental HD had significantly lower RKF loss than conventional HD [OR 0.31 (95% CI 0.25–0.39), *P* < .001] (Fig. 2).

Six studies [9, 12, 29, 31, 33, 37] evaluated the 24-hour urine output in incremental versus conventional HD. The change in urine output was significantly more preserved in incremental than conventional HD [MD 378 ml (95% CI 95–662 ml), *P* = .009].

### Intradialytic hypotension (IDH)

Three studies [13, 18, 35] reported the incidence of IDH. IDH was significantly lower in patients with incremental HD compared with conventional HD [OR 0.18 (95% CI 0.06–0.62), *P* = .006].

### Vascular access complications

Eight studies [12, 13, 15, 16, 26, 33, 36, 42] investigated the incidence of vascular access complications. There was no significant difference in vascular access complications in patients treated with incremental or conventional HD [OR 0.51 (95% CI 0.23–1.12), *P* = .091].

### Hyperkalaemia

Three studies [12, 15, 36] reported the incidence of hyperkalaemia. There was no significant difference in hyperkalaemia between patients who received incremental or conventional HD [OR 0.46 (95% CI 0.14–1.48), *P* = .192].

### Volume overload

Two studies examined the incidence of volume overload. Murea *et al.* [12] and Vilar *et al.* [15] reported no significant difference in volume overload between patients who received incremental or conventional HD.

### Hospitalization

**Figure 3:**
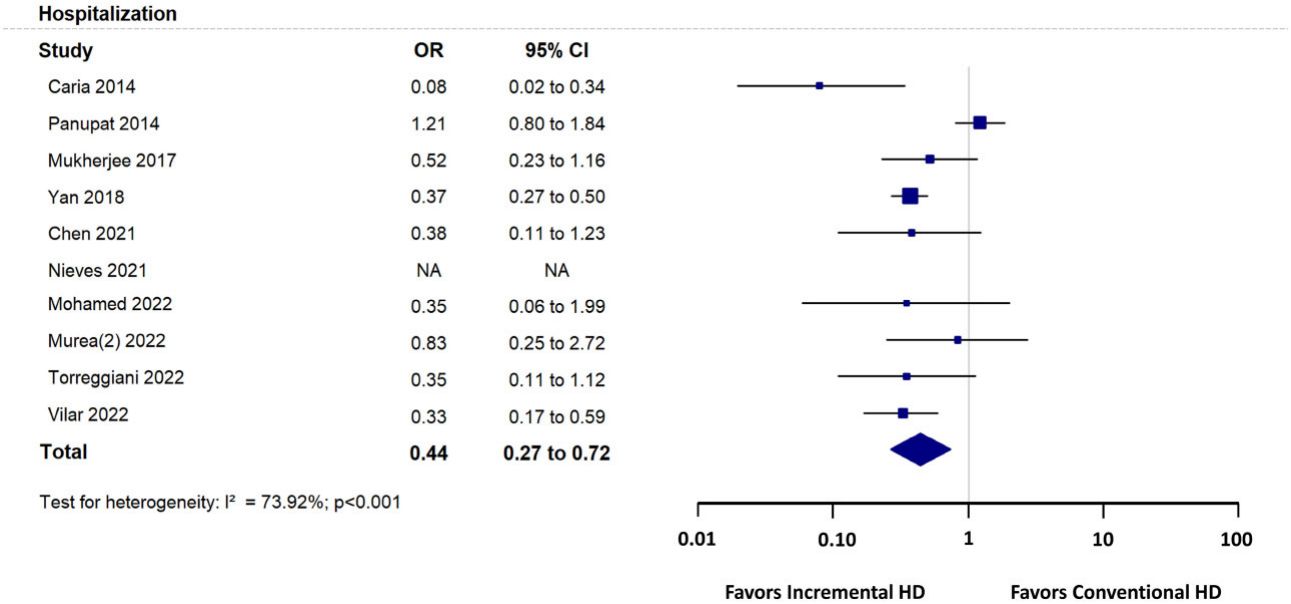
Hospitalization forest plot.

Ten studies [12–17, 25, 26, 33, 36] reported hospitalization. Hospitalization was significantly lower in patients treated with incremental compared with conventional HD [OR 0.44 (95% CI 0.27–0.72), *P* = 0.001] (Fig. 3).

### Cardiovascular events

Nine studies [13, 15, 19, 23, 26, 29, 33, 34, 36] determined the impacts of incremental versus conventional HD on cardiovascular events. There was no statistically significant difference in cardiovascular events between incremental and conventional HD [OR 0.67 (95% CI 0.43–1.05), *P* = .08]. Sensitivity analysis comparing studies of twice-weekly and conventional HD revealed no significant difference in cardiovascular events [OR 0.66 (95% CI 0.36–1.21), *P* = .178]. However, one study comparing once-weekly and conventional HD showed a significant difference [OR 0.11 (95% CI 0.01–0.95), *P* = .045] [33].

**Figure 4:**
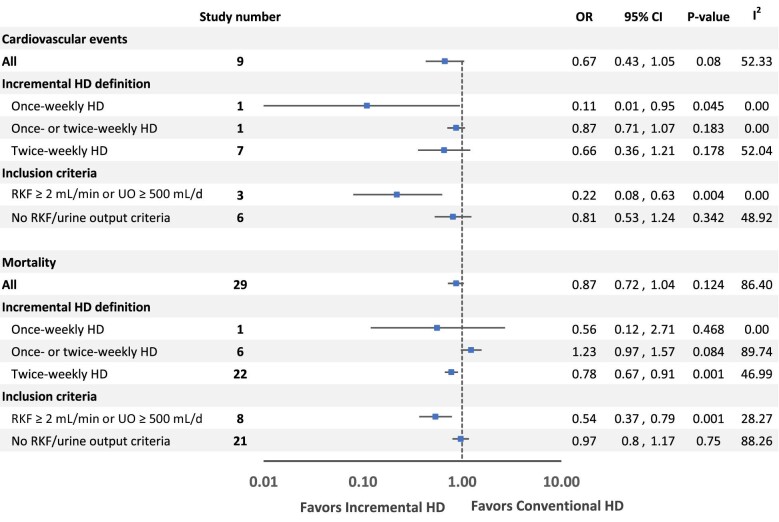
Subgroup analyses for cardiovascular events and mortality.

In three studies [13, 15, 33] that had RKF or urine output as criteria for initiating incremental HD, the cardiovascular events were significantly lower in patients with incremental HD compared with conventional HD [OR 0.22 (95% CI 0.08–0.63), *P* = .004] (Fig. 4).

### Mortality

**Figure 5:**
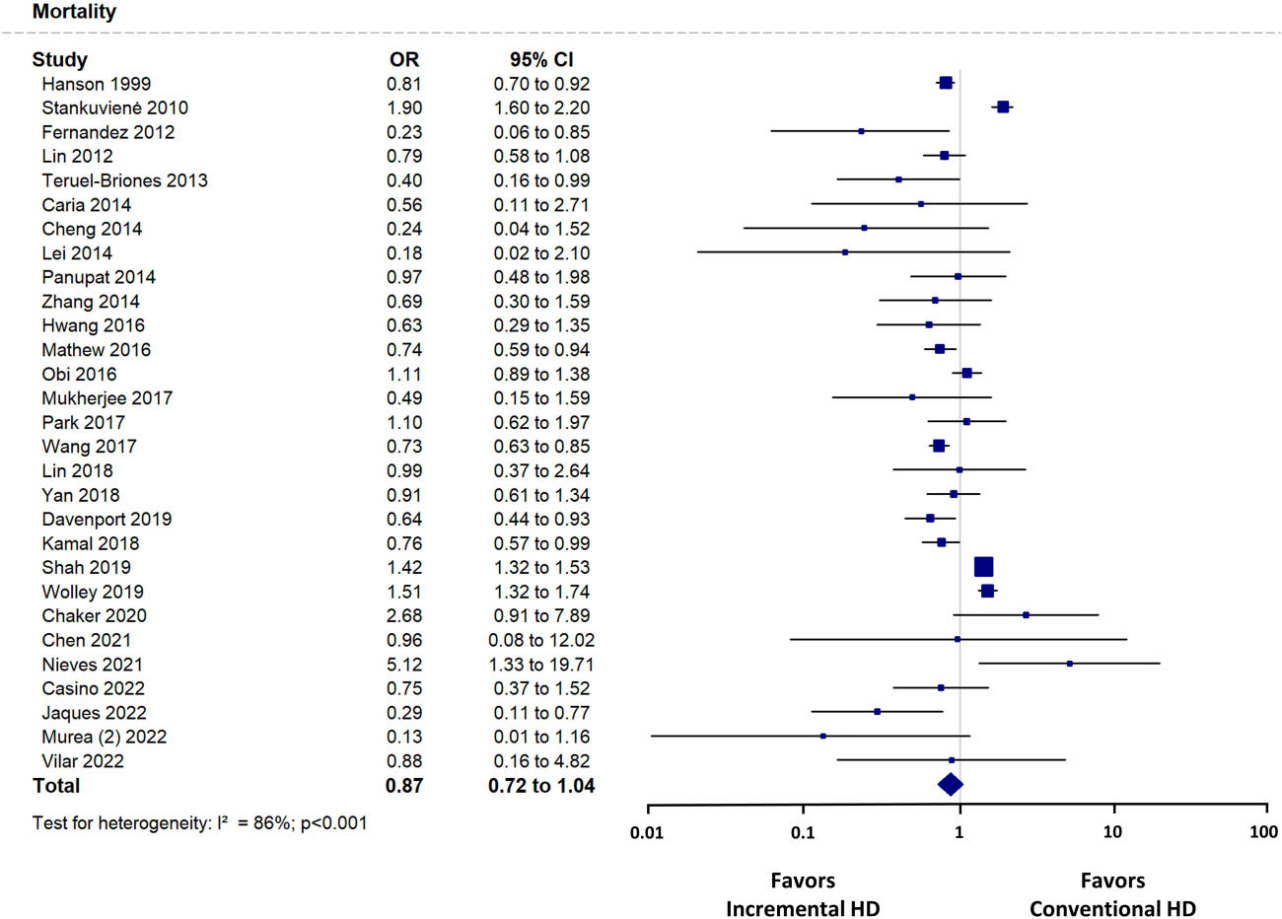
Mortality forest plot.

In a meta-analysis of 29 studies [9, 10, 12, 15–18, 20–31, 33–41, 44], there was no significant difference in mortality between patients treated with incremental or conventional HD [OR 0.87 (95% CI 0.72–1.04), *P* = .124] (Fig. 5). This finding was consistent across observational studies and RCTs. In sensitivity analyses, when we included only 22 studies [10, 12, 15, 17, 18, 20–22, 24–26, 28, 29, 31, 34–40, 44] that compared the mortality in twice-weekly versus conventional HD, the mortality was significantly lower in patients treated with twice-weekly HD than with thrice-weekly HD [OR 0.78 (95% CI 0.67–0.91), *P* = .001]. In addition, in eight studies [9, 10, 12, 15, 22, 33, 38, 39] that had RKF or urine output as criteria for initiating incremental HD, the mortality was significantly lower in patients with incremental HD compared with conventional HD [OR 0.54 (95% CI 0.37–0.79), *P* = .001].

### Quality of life

Four studies examined the impacts of incremental and conventional HD on quality of life. The data could not be combined into a meta-analysis due to the diversity of the quality-of-life assessments. Overall, there were no significant differences in the quality of life between incremental and conventional HD. Park *et al.* [27] revealed no significant difference in the Kidney Disease Quality of Life short form and Beck Depression Inventory (BDI) scores. Davenport *et al.* [20] reported no significant difference in BDI-II and nine-item Patient Health Questionnaire (PHQ-9) scores between the two groups. In two RCTs, Vilar *et al.* [15] did not find a significant difference in the quality of life and depression as measured by the EQ-5D-5L (EuroQol 5-dimension, 5-level) value and PHQ-9 score between the incremental and conventional HD groups. Murea *et al.* [11] observed no significant differences in PHQ-9 and Dialysis Symptom Index changes while revealing a significant reduction in anxiety using the seven-item Generalized Anxiety Disorder questionnaire in comparing conventional HD at 6 weeks.

### Publication bias

As Egger's test results were mainly non-significant (*P* > .05), together with a generally symmetrical funnel plot for the outcomes of the studies included in this meta-analysis, publication bias was less likely to occur (Supplementary Figs. S1–S4).

## DISCUSSION

The present meta-analysis explored the efficacy and safety of incremental versus conventional HD. Thirty-six articles were identified. We assessed the impacts on cardiovascular events, urine output, vascular access and HD complications, quality of life, hospitalization and mortality. We reported significantly lower hospitalization, loss of RKF and reduction of urine output in incremental versus conventional HD. Mortality and cardiovascular events were lower in incremental HD when the participants had RKF ≥2 ml/min or urine output ≥500 ml/day. Regarding safety, there were no significant differences in HD complications, including hyperkalaemia and volume overload.

RKF provides effective and continuous solute clearance and is associated with lower mortality in HD patients. Initiation with abruptly intense HD, particularly where significant ultrafiltration or intradialytic hypotension occurs, may lead to a rapid decrease in RKF. The concept of a gradual approach as incremental HD, derived from peritoneal dialysis, is expected to preserve RKF in HD patients. A previous meta-analysis revealed that urine volume was higher in patients on incremental HD than in those on conventional HD [45]. This finding is strengthened in our study, which included a larger number of studies and analysed the changes in urine output at baseline and the end of the study. We found that patients treated with incremental HD had less 24-hour urine output reduction and a lower RKF loss, defined by urine volume <200 ml/day, compared with conventional HD. It is important to note that the wide variability of RKF measurements among the studies, including urea and creatinine clearances, with or without normalization by body surface area, or reported in terms of the rate of RKF decline, leads to limitations in combining all available data for meta-analysis.

Other potential benefits of less frequent HD include reductions in vascular access problems and dialysis-associated complications such as IDH, myocardial ischaemia, arrhythmia and catheter-related infections. We found that patients undergoing incremental HD had a significantly lower incidence of IDH and a tendency towards lower rates of vascular access complications, although this difference was not statistically significant. Additionally, our results showed that incremental HD is associated with fewer hospitalizations. In support of the present study, Caton *et al.* [46] also reported reduced hospitalization rates in patients receiving incremental HD compared with conventional HD.

In the present meta-analysis, overall mortality did not differ significantly between incremental and conventional HD, as discovered in the previous meta-analysis. However, participants in some studies received less frequent HD due to financial problems or lack of adequate healthcare services and therefore were not the optimal candidates for incremental HD. According to the Kidney Disease Outcomes Quality Initiative Clinical Practice Guidelines for Hemodialysis Adequacy [47], patients with urea clearance <2 ml/min should undergo HD three times per week. We found that in studies in which participants had RKF of at least 2 ml/min or urine output ≥500 ml/day, incremental HD had significantly fewer cardiovascular events and mortality than conventional HD. Furthermore, complications associated with inadequate dialysis, including hyperkalaemia and volume overload, were not different between incremental and thrice-weekly HD. These findings emphasize the importance of patient selection and RKF measurement in the incremental HD approach. However, due to the variations in RKF between patients undergoing incremental and conventional HD in these studies, it is important to note that better outcomes in patients with incremental HD who have RKF might not result solely from a different HD prescription, but could also be influenced by the presence of RKF, which is known as an important factor associated with survival [48]. In addition, when we excluded the studies that enrolled patients in once-weekly HD, we discovered that twice-weekly HD was associated with lower mortality than thrice-weekly HD. It is possible that the lack of strict requirements for RKF and regular monitoring in several studies that administered once-weekly HD may have contributed to suboptimal HD and poor outcomes. Implementing appropriate once-weekly HD remains challenging and insufficient data are available. Few reports described the cohorts or experience in a single centre with the successful application of once-weekly HD [49, 50]. Further work should be conducted through well-designed RCTs with optimal selection criteria to investigate the feasibility, safety and efficacy of once-weekly HD.

We observed that standardized *Kt*/*V* was lower in patients undergoing the incremental approach compared with those receiving thrice-weekly HD. However, there were no significant differences in serum haemoglobin, phosphate and hyperkalaemia levels between the two groups. In addition, patients treated with incremental HD had significantly lower levels of serum β2M than those on conventional HD. This could be due to better RKF preservation in incremental HD and may contribute to fewer cardiovascular events and lower mortality in this group [51]. Our results also showed that while patients undergoing incremental HD had lower protein intake as measured by a lower nPCR compared with those on conventional HD, there was no significant difference in their serum albumin levels. It is important to consider the appropriate level of protein intake for patients on incremental HD, as excess protein can lead to impaired renal function while insufficient protein intake can cause malnutrition. Further research should be conducted to determine the optimal protein intake in this population.

An improvement in quality of life, both physical and mental, is another possible benefit of incremental HD. Common complaints that can negatively impact the patient's quality of life, including prolonged transit time to and from the dialysis centre, intradialytic symptoms and the recovery period post-dialysis, may be alleviated by applying a less frequent dialysis regimen [52]. However, assessments of the quality of life, anxiety and depression of patients were based on a variety of scores and were difficult to combine or compare across the studies. Most of the studies reported insignificant impacts of incremental HD on quality of life compared with conventional HD. Of note, one RCT demonstrated the superiority of incremental HD over conventional HD in reducing anxiety. The impacts of incremental HD on quality of life should be explored in future studies.

There are some limitations in our study. First, the decision to prescribe incremental or conventional HD varied among the included studies and may contribute to heterogeneity in the results. Second, variations in outcome definition and the duration of follow-up may confound the analysis. Lastly, it is important to note that the majority of the included studies are observational, which may introduce limitations in terms of bias and the interpretation of causality. Currently, several RCTs have been registered for the protocol and are being conducted to determine the efficacy and impact of incremental HD [53–56].

## CONCLUSIONS

Current evidence demonstrates that incremental HD is safe and potentially provides reductions in hospitalization and loss of RKF compared with conventional HD. In appropriately selected patients, incremental HD may improve cardiovascular outcomes and reduce mortality. Further well-designed RCTs are needed to fully assess the efficacy and impact of incremental HD compared with conventional HD and to determine whether more frequent than twice-weekly HD should remain the standard of care.

## Supplementary Material

sfad280_Supplemental_File

## Data Availability

The data underlying this article will be shared upon reasonable request to the corresponding author.
