# Supplementary material for: Incremental versus conventional haemodialysis in end-stage kidney disease: a systematic review and meta-analysis
Source: Clin Kidney J. 2023 Nov 8;17(1):sfad280. doi: 10.1093/ckj/sfad280 (PMC10768771; doi:10.1093/ckj/sfad280)
Supplement: sfad280_Supplemental_File [file sfad280_supplemental_file.pdf]

Funnel Plot of Standard Error by Log odds ratio (Mortality)

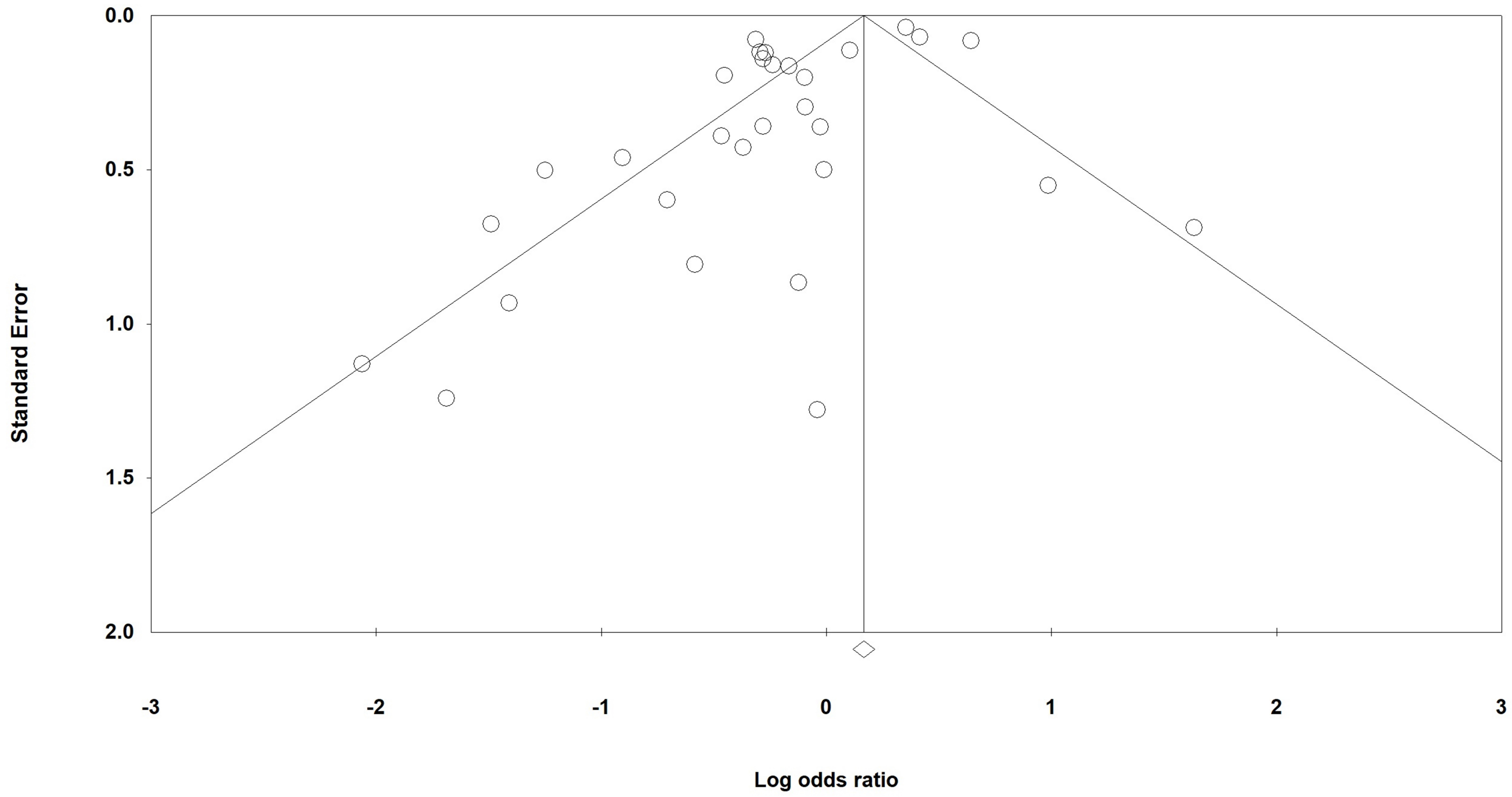

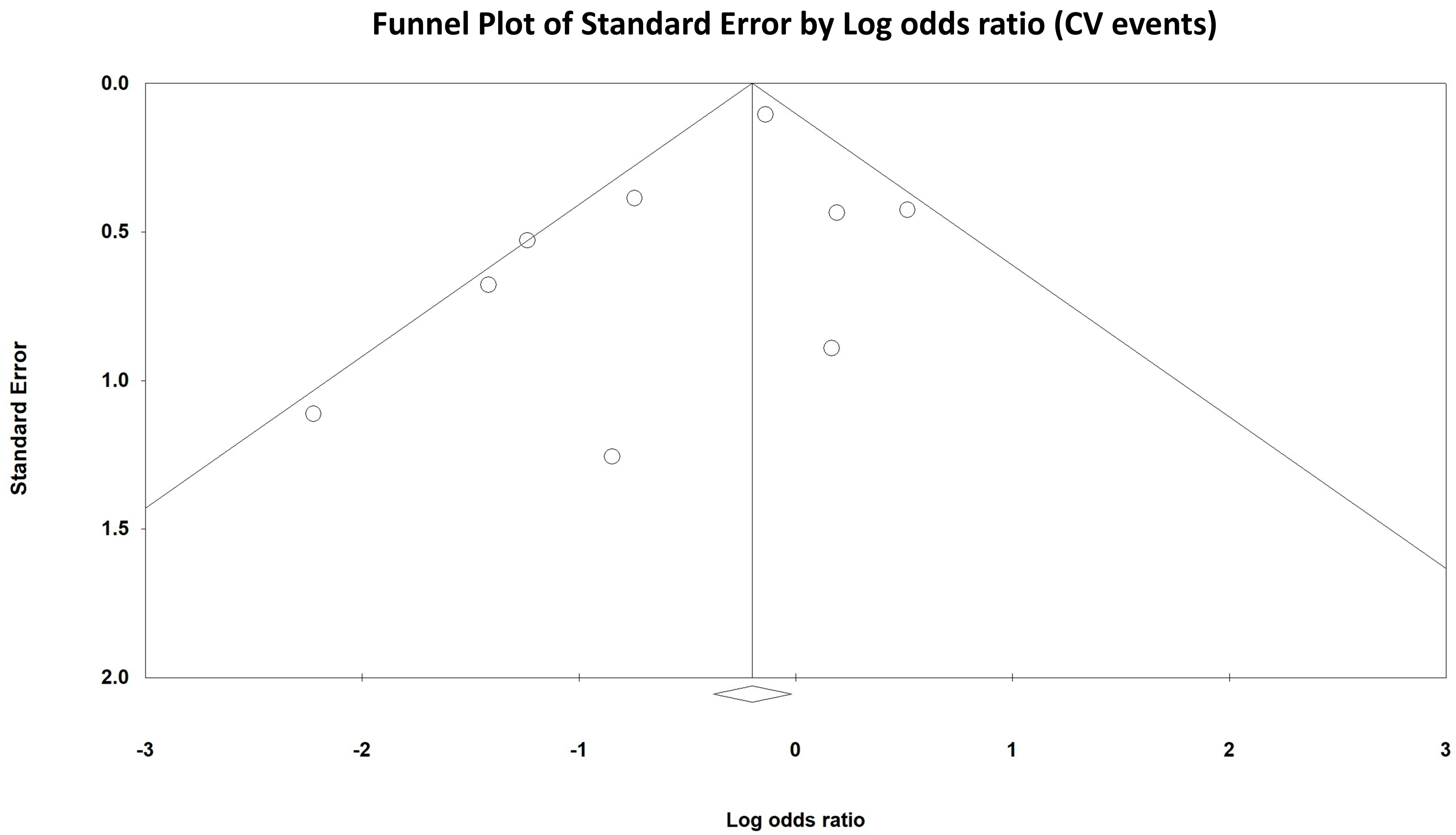
Funnel Plot of Standard Error by Log odds ratio (CV events)
0.0
0.5
1.0
1.5
2.0
Standard Error
-3
-2
-1
0
1
2
3
Log odds ratio

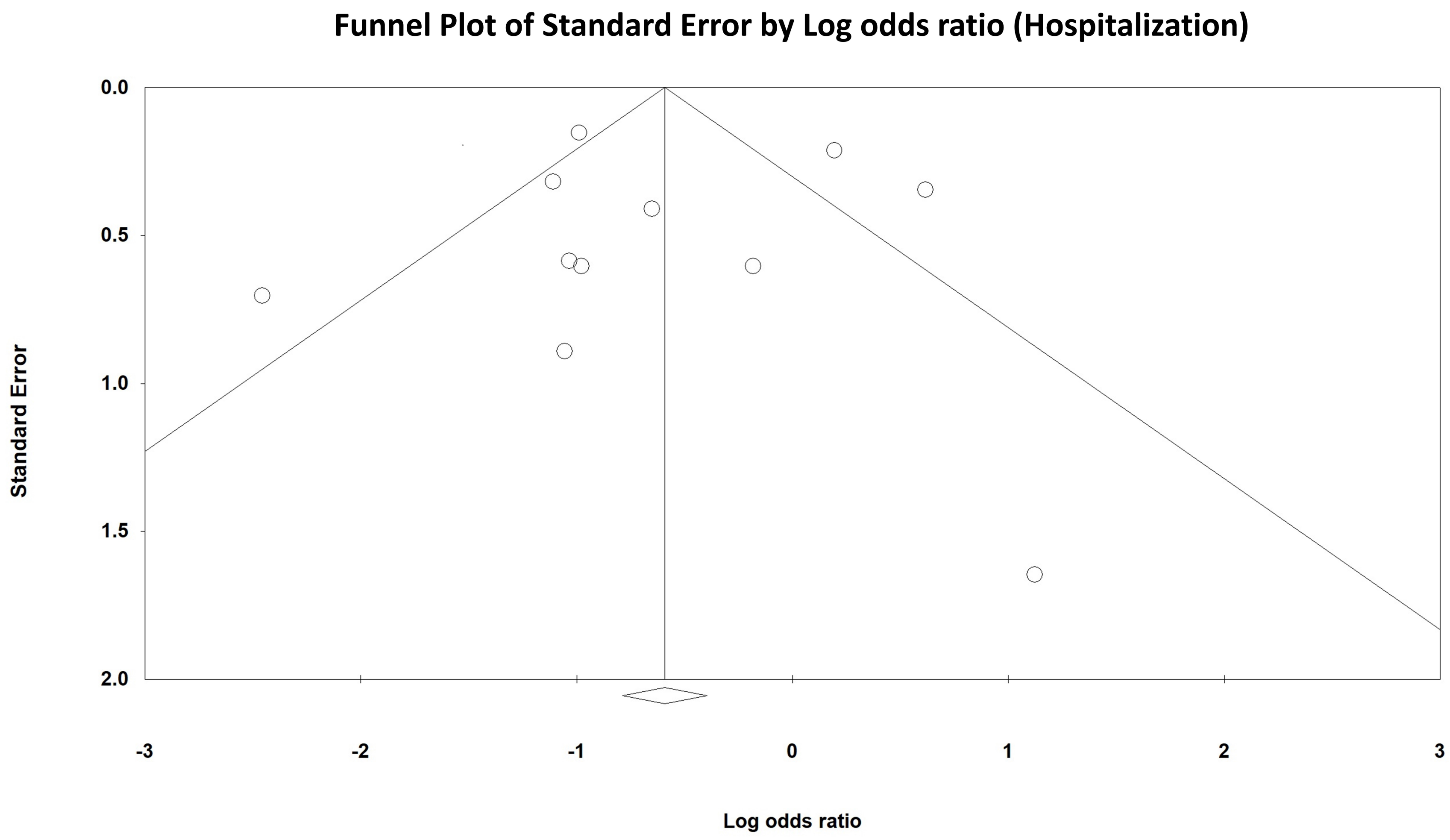
Funnel Plot of Standard Error by Log odds ratio (Hospitalization)
0.0
0.5
1.0
1.5
2.0
Standard Error
-3
-2
-1
0
1
2
3
Log odds ratio

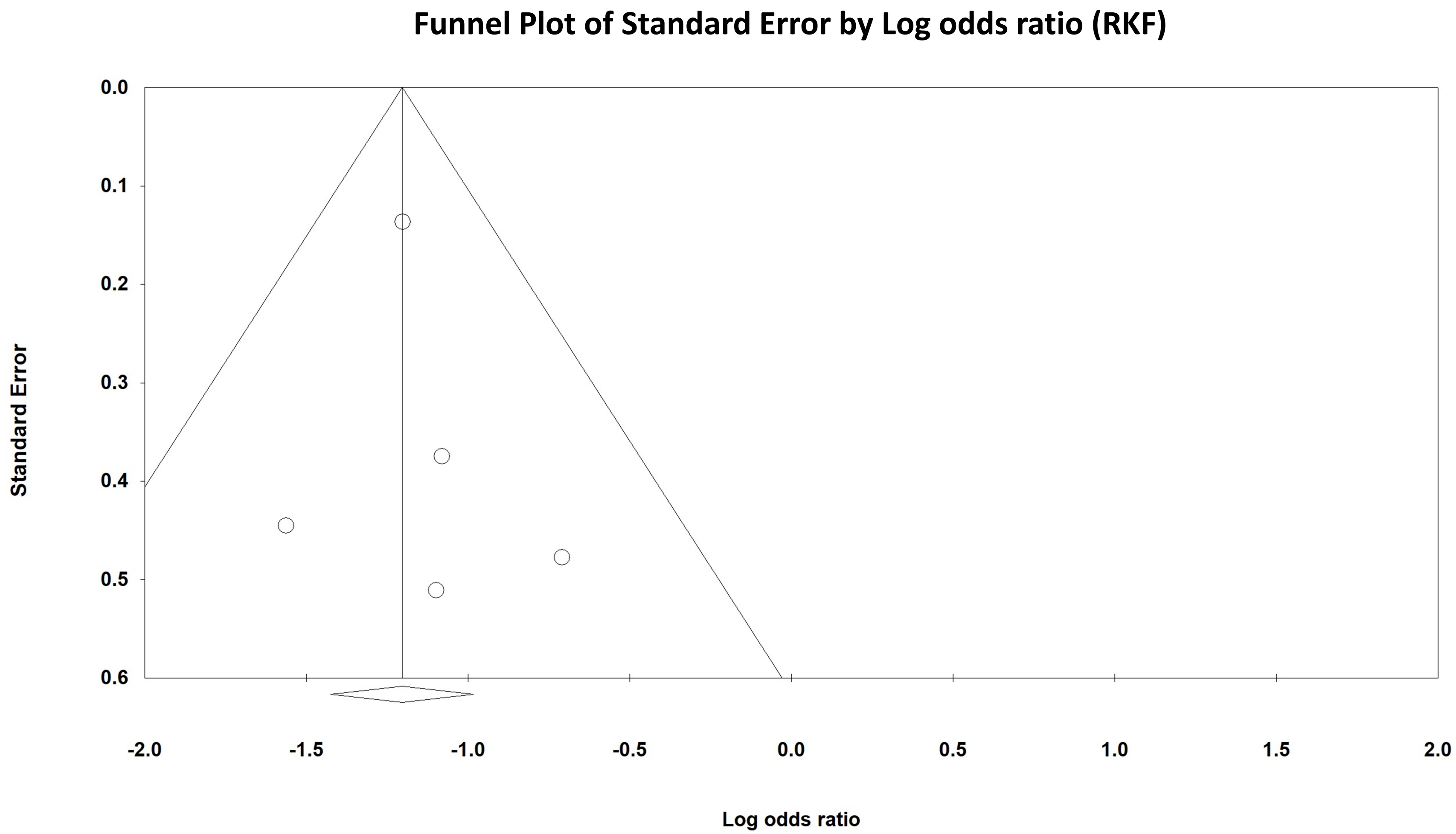
Funnel Plot of Standard Error by Log odds ratio (RKF)
Standard Error
0.0
0.1
0.2
0.3
0.4
0.5
0.6
-2.0
-1.5
-1.0
-0.5
0.0
0.5
1.0
1.5
2.0
Log odds ratio
